# Small secreted proteins from the necrotrophic conifer pathogen *Heterobasidion annosum s.l*. (HaSSPs) induce cell death in *Nicotiana benthamiana*

**DOI:** 10.1038/s41598-017-08010-0

**Published:** 2017-08-11

**Authors:** Tommaso Raffaello, Fred O. Asiegbu

**Affiliations:** Department of Forest Sciences, University of Helsinki, Faculty of Agriculture and Forestry, Latokartanonkaari 7, 00014 Helsinki, Finland

## Abstract

The basidiomycete *Heterobasidion annosum* sensu lato (*s.l*.) is considered to be one of the most destructive conifer pathogens in the temperate forests of the northern hemisphere. *H*. *annosum* is characterized by a dual fungal lifestyle. The fungus grows necrotrophically on living plant cells and saprotrophically on dead wood material. In this study, we screened the *H*. *annosum* genome for small secreted proteins (HaSSPs) that could potentially be involved in promoting necrotrophic growth during the fungal infection process. The final list included 58 HaSSPs that lacked predictable protein domains. The transient expression of HaSSP encoding genes revealed the ability of 8 HaSSPs to induce cell chlorosis and cell death in *Nicotiana benthamiana*. In particular, one protein (HaSSP30) could induce a rapid, strong, and consistent cell death within 2 days post-infiltration. HaSSP30 also increased the transcription of host-defence-related genes in *N. benthamiana*, which suggested a necrotrophic-specific immune response. This is the first line of evidence demonstrating that the *H. annosum* genome encodes HaSSPs with the capability to induce plant cell death in a non-host plant.

## Introduction

The basidiomycete *Heterobasidion annosum* sensu lato (*s.l*.) is a necrotrophic fungal pathogen of conifer trees^1^. *H. annosum s.l*. exists as a species complex with three European species, *H. annosum* sensu stricto (*s.s*.), *H. parviporum*, and *H. abietinum*, and two north American species, *H. irregulare* and *H. occidentale* ^2^. While *H. abietinum* is found mostly in the Mediterranean area, infecting conifer species of the genus *Abies*, *H. parviporum* and *H. annosum s.s*. are found in northern Europe, infecting mainly Norway spruce and Scots pine, respectively^3, 4^. The *H. annosum s.l*. initially infects hosts through stump colonization mediated by basidiospores. The fungus from infected trees spreads to healthy trees by root-to-root contact^5^. The fungal infection compromises the timber quality, causing an estimated economic loss of approximately 790 million euros per year in Europe alone^4^. A major feature of this pathogen is the ability to switch from a saprotrophic (i.e., feeding on wood material) to a necrotrophic lifestyle when it encounters living cells in the sapwood of infected conifer trees.

In nature, forest trees and crop plants are constantly challenged by a wide variety of pathogens, including fungi, oomycetes, viruses, and bacteria. Because plants are sessile organisms unable to evade the attacker by simply moving away, they have developed a sophisticated immune system. Plants respond to microbial invaders by coordinating the actions of several elements of the plant immune system^6^. In the classic plant-microbe interaction model, invading pathogens possess pathogen associated molecular patterns (PAMPs) which are recognized by specific plant pattern recognition receptors (PRRs); the recognition between a PAMP and its cognate PRR triggers an immune response defined as PAMP-triggered immunity (PTI). However, the PTI response is attenuated by the secretion of microbial effectors that suppress the plant immune system to promote host colonization^6^. The highly specific recognition between a plant resistance protein (R protein) and the pathogen effector follows the gene-for-gene interaction hypothesis and culminates with the plant’s programmed cell death (PCD) or hypersensitive response (HR) mechanisms^7^.

Plant pathogenic fungi are divided primarily into biotrophs, hemibiotrophs, and necrotrophs based on their mode of infection and host colonization. Biotrophs are so defined because they feed on living plant tissue. In contrast, necrotrophs rapidly kill the host tissue and feed on dead plant material. Hemibiotrophs are similar to both in that they are characterized by an initial biotrophic phase followed by a switch to a necrotrophic phase during late infection.

Fungal plant pathogens colonize the host by secreting a wide array of fungal effectors^8^. Most of the research related to the functional characterization of fungal effectors has been performed using biotrophic and hemibiotrophic fungi. Since the first fungal plant pathogen genome (*Magnaporthe oryzae*) was sequenced^9^, several studies have been published elucidating the mechanisms of action of biotrophic fungal effectors. For example, several fungal effector proteins bind and mask chitin to prevent triggering the plant immune response. Examples of these effectors are as follows: *Cladosporium fulvum* Ecp6, which binds chitin oligomers^10^; *C. fulvum* Avr4, which masks the chitin of the fungal cell wall to protect it from the plant chitinases^11^; and *M. oryzae* Slp1, which has chitin binding properties^12^. Other fungal effectors can target and inhibit the cellular proteases that are important for the plant immune response. For example, *U. maydis* Pit2 and *C. fulvum* Avr2 bind and inhibit several plant cysteine-proteases^13, 14^.

Very little is known about the mechanisms of action of necrotrophic fungal effectors. Necrotrophic fungi have historically been considered unspecialized and unsophisticated plant pathogens that can only secrete cell wall degrading enzymes (CWDE) to induce nonspecific cell death by compromising plant cell wall integrity^15^. However, several lines of evidence demonstrate that necrotrophic fungi can produce and secrete specific proteins that specifically interact with components of the plant immune system. In particular, necrotrophic fungi can secrete host specific toxins (HST) that are defined as necrotrophic effectors^16, 17^. Much of what we know about these effectors stems from studies characterizing the crop pathogens *Stagonospora nodorum* and *Pyrenophora tritici-repentis*, which are two necrotrophic fungi of wheat responsible for the Stagonospora nodorum blotch (SNB) and tan spot, respectively^17^. The first HST to be characterized was ToxA, a pathogenicity factor of *P. tritici-repentis*, which is responsible for the fungal necrotrophic growth in wheat (*Triticum aestivum*)^18^. There is much less literature describing necrotrophic effectors than biotrophic parasites, and most available information is primarily related to agricultural crop pathogens. Additionally, the data available on necrotrophic effectors for forest pathogenic fungi is limited^19, 20^.

To date, effectors for the necrotrophic fungus *H. annosum s.l*. have not been identified. The availability of the sequenced and annotated genome of *H. annosum* strain TC32-1 provided the opportunity to screen for putative fungal necrotrophic effectors^21^. This study provides the first line of evidence that the *H. annosum* genome encodes small secreted proteins (HaSSPs) with the ability to induce plant cell death in the plant model system *N. benthamiana*.

## Results

### Genome mining for candidate *H. annosum* small secreted protein (HaSSP)- encoding genes and assessment of gene expression

**Figure 1:**
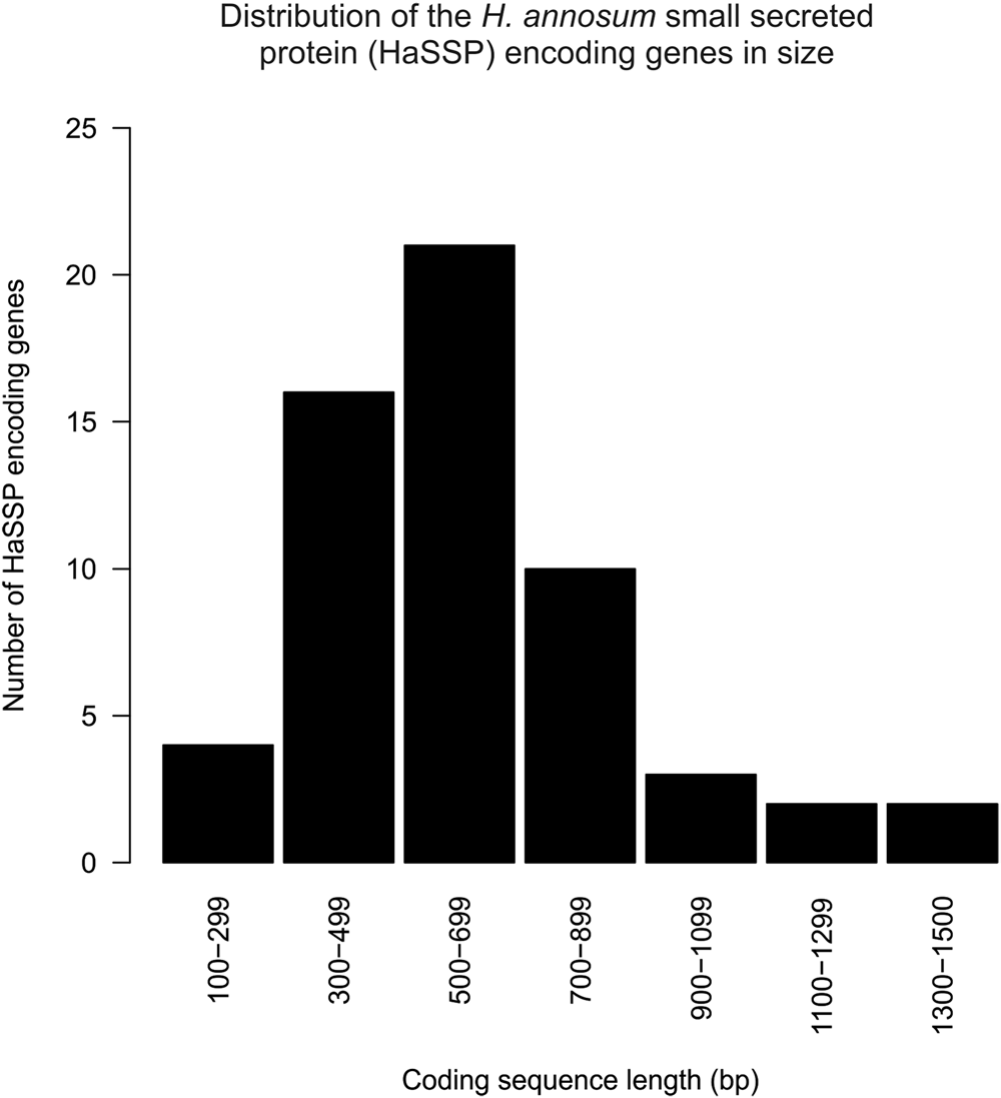
Distribution of the length of the *H. annosum* small secreted protein (HaSSP) encoding genes as retrieved from the *H. annosum* TC 32-1 genome annotation.

Fifty-eight putative HaSSP encoding genes were retrieved from the genome of *H. annosum* strain TC 32-1 (Table 1). The shortest candidate gene was 174 nucleotides (nt) long [Protein ID (Hetan2) 328808, 58 AA], while the longest was 1428 nt long [Protein ID (Hetan2) 441917, 476 AA]. The length of most of the candidate effector genes was between 300 nt and 900 nt (Fig. 1), and the HaSSP proteins selected for this study did not include any predicted protein domains. The number of disulfide bridges ranged between 1 and 8 [Protein ID (Hetan2) 482211, 442 AA]. Twenty-three HaSSP-encoding genes were characterized by expressed sequence tags (EST) support in the *H. annosum* TC 32-1 genome browser (Table 1).

**Table 1:** Summary of the *H. annosum* small secreted protein (HaSSP) encoding genes retrieved from the *H. annosum* TC 32-1 genome annotation.

HaSSP candidate number	Protein ID (Hetan1)^1^	Protein ID (Hetan2.0)^1^	Gene name (Hetan2)^1^	Length (bp)	Length (AA)	Protein ID	Cys-Cys bonds number	EST support
1	58748	58748	Hetan1.estExt_Genewise1Plus.C_10476	585	195	58748	5	YES
2	99056	99056	Hetan1.Genemark.129_g	900	300	99056	5	NO
3	101504	101504	Hetan1.Genemark.2577_g	468	156	101504	1	NO
4	102625	102625	Hetan1.Genemark.3698_g	351	117	102625	1	NO
5	102999	102999	Hetan1.Genemark.4072_g	615	205	102999	2	NO
6	103544	103544	Hetan1.Genemark.4617_g	582	194	103544	2	NO
7	106199	106199	Hetan1.Genemark.7272_g	645	215	106199	2	NO
8	106204	106204	Hetan1.Genemark.7277_g	339	113	106204	1	NO
9	107400	107400	Hetan1.Genemark.8473_g	387	129	107400	1	NO
10	107522	107522	Hetan1.Genemark.8595_g	483	161	107522	2	NO
11	108275	108275	Hetan1.Genemark.9348_g	1233	411	108275	4	NO
12	108527	108527	Hetan1.Genemark.9600_g	432	144	108527	3	NO
13	109064	109064	Hetan1.Genemark.10137_g	558	186	109064	5	YES
14	118007	118007	Hetan1.fgenesh2_pg.C_scaffold_7000137	744	248	118007	1	YES
15	119220	119220	Hetan1.fgenesh2_pg.C_scaffold_10000169	822	274	119220	2	NO
16	120415	120415	Hetan1.fgenesh2_pg.C_scaffold_13000272	741	247	120415	2	NO
17	120417	120417	Hetan1.fgenesh2_pg.C_scaffold_13000274	453	151	120417	1	NO
18	120417	120798	Hetan1.fgenesh2_pg.C_scaffold_15000086	606	202	120798	1	NO
19	157644	157644	Hetan1.estExt_fgenesh2_pm.C_130199	453	151	157644	2	YES
20	162925	162925	Hetan1.EuGene10000530	222	74	162925	2	NO
21	163365	163365	Hetan1.EuGene11000336	306	102	163365	3	NO
22	173217	173217	Hetan1.EuGene7000278	327	109	173217	5	YES
23	174683	174683	Hetan1.EuGene9000335	273	91	174683	4	NO
24	116393	312085	e_gw1.03.1544.1	618	206	312085	2	NO
25	34813	314110	e_gw1.03.1702.1	471	157	314110	3	YES
26	104923	317287	e_gw1.05.2448.1	1092	364	317287	2	NO
27	25033	318819	e_gw1.05.2082.1	798	266	318819	1	NO
28	55873	326012	e_gw1.09.1661.1	720	240	326012	4	NO
29	NA	328808	e_gw1.11.1302.1	174	58	328808	3	NO
30	164295	391204	estExt_Genewise1.C_131090	534	178	391204	1	YES
31	NA	414262	fgenesh1_pm.02_#_149	417	139	414262	3	YES
32	115627	418080	fgenesh1_pm.05_#_676	372	124	418080	2	NO
33	169544	418760	fgenesh1_pm.06_#_505	336	112	418760	4	YES
34	162746	422598	fgenesh1_pm.12_#_283	492	164	422598	3	NO
35	108551	423383	fgenesh1_pm.14_#_116	378	126	423383	2	NO
36	116836	425913	fgenesh1_pg.03_#_659	813	271	425913	4	NO
37	NA	425924	fgenesh1_pg.03_#_670	891	297	425924	2	NO
38	154711	427282	fgenesh1_pg.05_#_541	795	265	427282	NA	YES
39	115568	427322	fgenesh1_pg.05_#_581	726	242	427322	2	NO
40	152005	429704	fgenesh1_pg.10_#_299	525	175	429704	3	NO
41	120216	430810	fgenesh1_pg.13_#_214	507	169	430810	1	NO
42	145189	431393	estExt_fgenesh1_pm.C_010330	798	266	431393	1	YES
43	108765	439412	estExt_fgenesh1_kg.C_040024	306	102	439412	2	YES
44	148537	439593	estExt_fgenesh1_kg.C_040240	696	232	439593	1	YES
45	147795	441917	estExt_fgenesh1_kg.C_100319	1428	476	441917	4	YES
46	148061	442002	estExt_fgenesh1_kg.C_110095	603	201	442002	5	YES
47	NA	447006	estExt_fgenesh1_pg.C_130285	660	220	447006	1	YES
48	157214	450757	Genemark.3585_g	582	194	450757	1	NO
49	104830	451269	Genemark.4097_g	642	214	451269	4	NO
50	103839	452658	Genemark.5486_g	627	209	452658	2	NO
51	103483	452993	Genemark.5821_g	537	179	452993	3	YES
52	NA	456226	Genemark.9054_g	618	206	456226	2	YES
53	146303	459335	estExt_Genemark.C_060269	216	72	459335	4	YES
54	108486	461461	estExt_Genemark.C_140166	1011	337	461461	4	NO
55	168056	470877	estExt_Genewise1Plus.C_021603	252	84	470877	2	YES
56	104047	477946	estExt_Genewise1Plus.C_080375	465	155	477946	2	YES
57	107965	482211	estExt_Genewise1Plus.C_130878	1326	442	482211	8	YES
58	165012	482547	estExt_Genewise1Plus.C_140371	627	209	482547	4	YES

^1^ http://genome.jgi.doe.gov/Hetan2/Hetan2.home.html.

**Figure 2:**
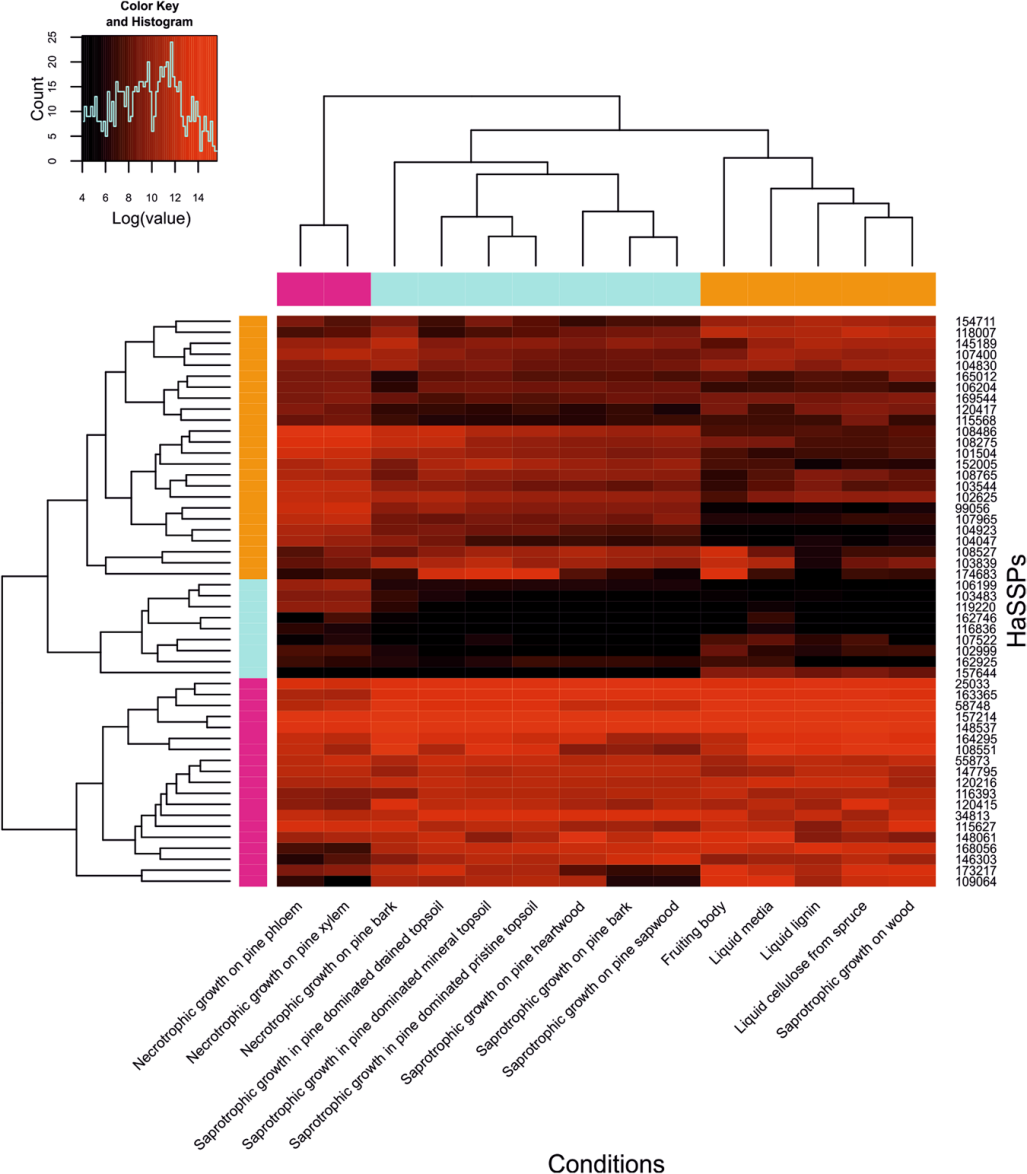
Microarray gene expression levels of the selected *H. annosum* small secreted protein (HaSSP) encoding genes in different conditions. Microarray data were retrieved from the Gene Expression Omnibus database (GEO) (https://www.ncbi.nlm.nih.gov/geo/). Raw data were normalized and analysed with the statistical R programme^48^ using *oligo* ^49^ and *gplots* ^50^ packages.

The transcriptomic data available for *H. annosum* in the GEO database were collected from samples grown under different conditions and substrates. These data revealed that the selected HaSSP-encoding genes displayed a characteristic variation in their expression levels (Fig. 2). In particular, sample cluster analysis revealed a single separated cluster for the samples related to the fungal necrotrophic growth on pine phloem and xylem (“Necrotrophic growth on pine phloem” and “Necrotrophic growth on pine xylem”, pink cluster, Fig. 2). All the samples related to saprotrophic growth on pine sapwood, heartwood and bark, together with the sample related to necrotrophic growth on pine bark, formed a separate cluster (light blue cluster, Fig. 2). Finally, all the samples related to growth on liquid media together with the samples from the *H. annosum* fruiting body and saprotrophic growth on whole wood formed another separate cluster (orange cluster, Fig. 2). The three clusters described above were statistically significant, as shown by the multiscale bootstrap resampling analysis (Supplementary Fig. S1). The multiscale bootstrap resampling analysis revealed approximately unbiased (*au*) values of 100, 96, and 92 for the necrotrophic, saprotrophic, and liquid clusters, respectively (Supplementary Fig. S1).

The cluster analysis of the HaSSP-encoding genes revealed three main transcript groups. The first cluster was characterized by high gene expression levels (pink cluster, Fig. 2), the second was characterized by low gene expression levels (light blue cluster, Fig. 2), and finally, the third was characterized by intermediate gene expression levels (orange cluster, Fig. 2). The three gene clusters were also statistically significant as shown by the multiscale bootstrap resampling analysis (Supplementary Fig. S2). The analysis revealed an approximately unbiased (*au*) value of 89, 97, and 97 for the high, low and intermediate gene expression level clusters, respectively (Supplementary Fig. S2).

### Transient expression of the selected *H. annosum* small secreted protein (HaSSP)- encoding genes in *Nicotiana benthamiana* by agroinfiltration

**Figure 3:**
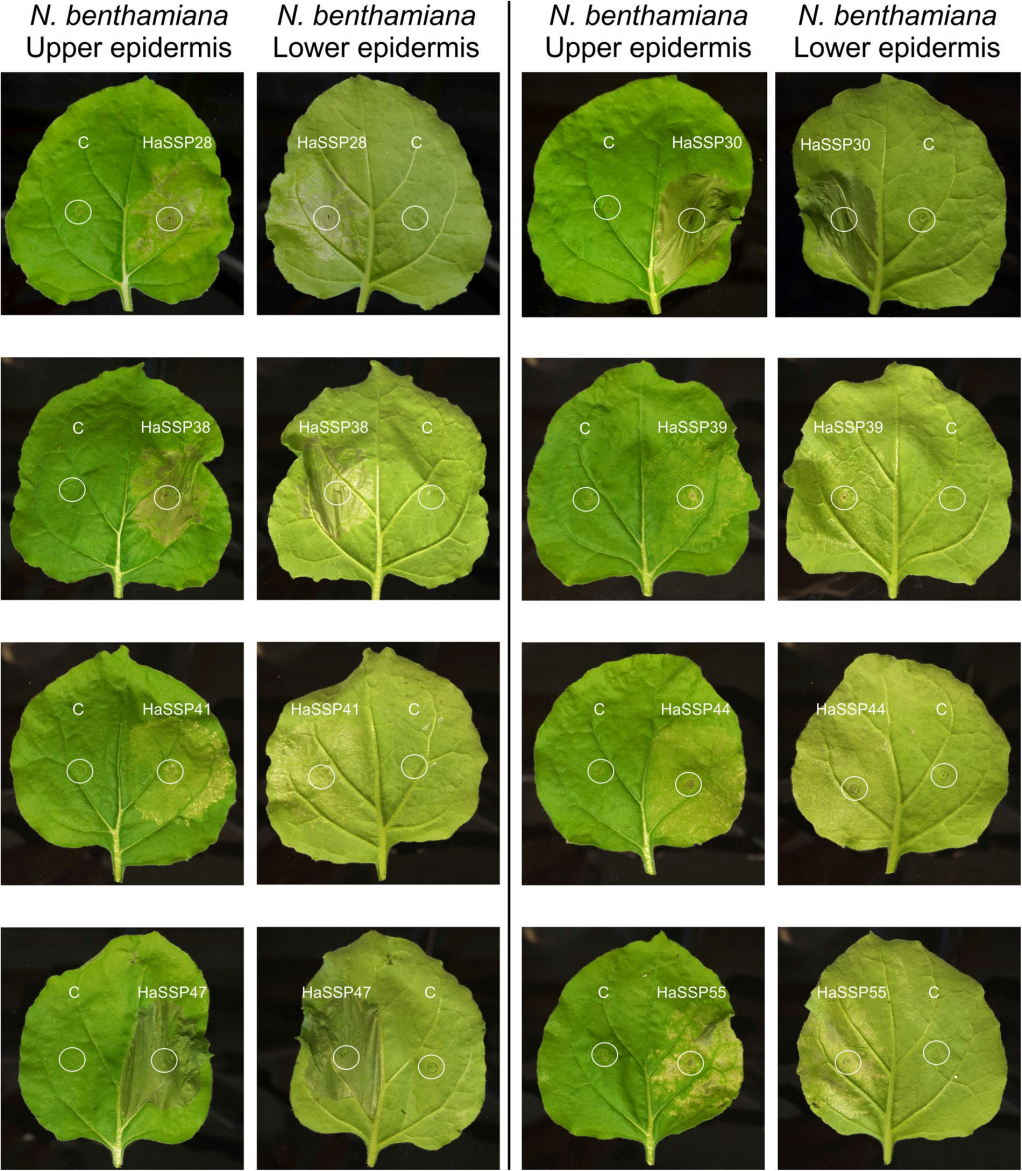
Chlorosis and cell death induced by transient expression of the *H. annosum* small secreted protein (HaSS) encoding genes in *Nicotiana benthamiana*. HaSSPs were custom synthesised into the pUC57 vector. Each candidate gene was cloned into the pICH86988 expression vector by Golden Gate cloning, generating pICH86988-HaSSP. The vector was introduced into *Agrobacterium tumefaciens* GV3101 followed by agroinfiltration into *Nicotiana benthamiana* leaves. The plants were incubated in a growth room with 12 h/12 h, night/day photoperiods at 20–24 °C and 60% relative humidity (RH). The infiltrated leaves were monitored for 2 to 6 days post-infiltration (dpi).

Eight constructs out of the 58 *H. annosum* HaSSP-encoding genes tested in this study induced chlorosis and cell death when transiently expressed in *N. benthamiana* by agroinfiltration (Fig. 3). Plant cell death was characterized by large necrotic spots, thinning, and overall compromised tissue integrity compared to the control, which showed no symptoms. In particular, HaSSP28, HaSSP38, HaSSP39, HaSSP41, HaSSP44, and HaSSP55 induced a certain level of chlorosis and cell death between 3 to 6 days post-infiltration (dpi). However, HaSSP30 and HaSSP47 induced a rapid and more pronounced cell death as early as 2 dpi and total loss of leaf turgidity at 4 dpi. Following sequence analysis, HaSSP30 and HaSSP47 were found to represent two different gene isoforms (ID391204 and ID447006 respectively, Table 1) but denoted the same protein predicted in the *H. annosum* TC 32-1 genome. The two predicted proteins only differed due to an extra fragment of 45 amino acids at the C-terminus of HaSSP47 (Supplementary Fig. S3).

### Activation of selected *N. benthamiana* immune system genes by HaSSP30

**Figure 4:**
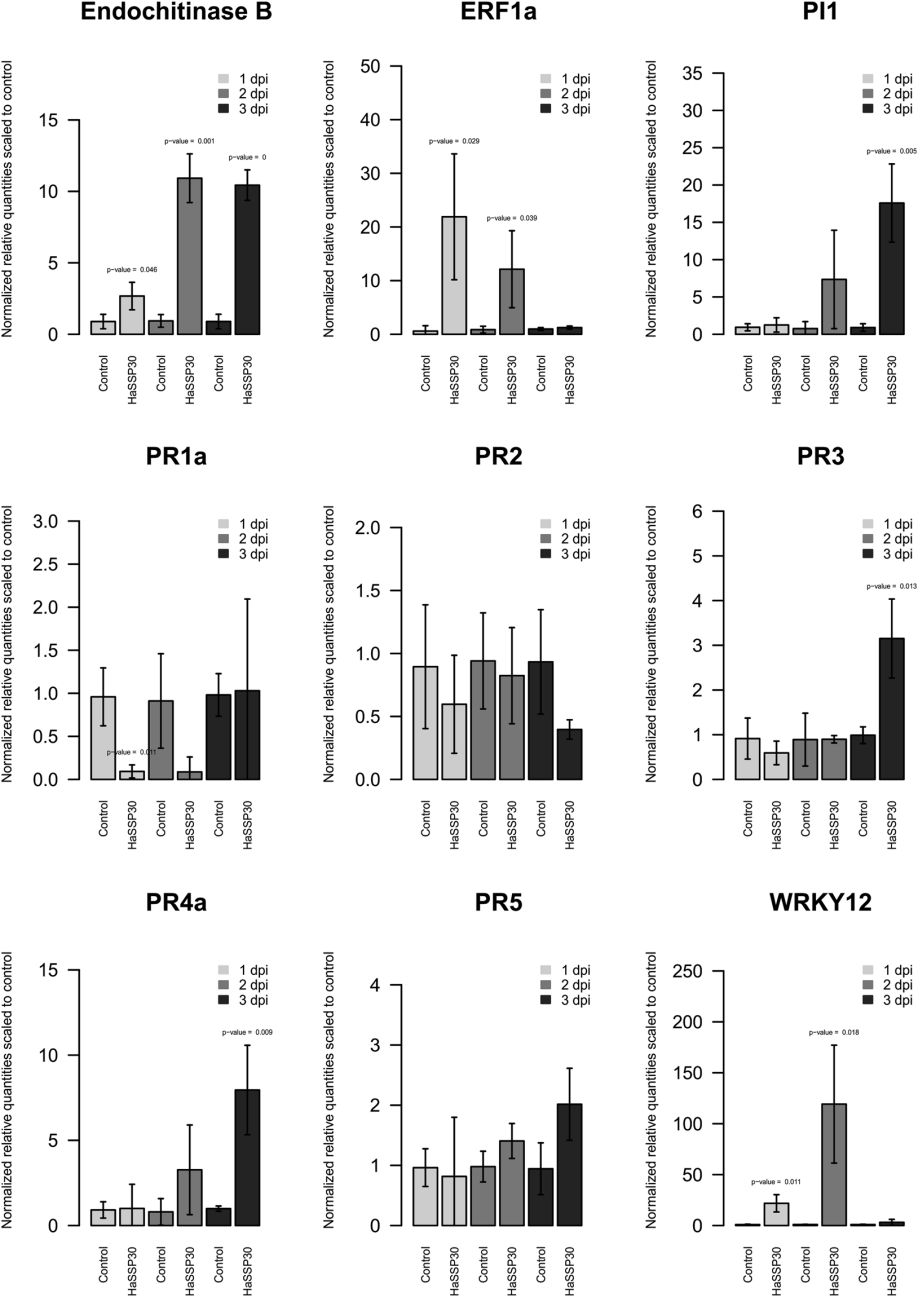
Gene expression levels of several markers related to plant immunity in *Nicotiana benthamiana* leaves transiently expressing HaSSP30 for 1, 2, and 3 days. *Nicotiana benthamiana* leaves were infiltrated with *Agrobacterium tumefaciens* GV3101 carrying either the pICH86988-empty (Control) or pICH86988-HaSSP30 (HaSSP30) vector. The plants were incubated in a plant growth room with 12 h/12 h, night/day photoperiods at 20–24 °C with 60% relative humidity (RH). The leaves were harvested after 1, 2, and 3 days post-infiltration (dpi). Total RNA was extracted and reverse transcribed into cDNA. The qPCR was performed on the cDNA using specific primers for several genes related to activation of plant immunity. Two reference genes were used to normalize the data, which are presented as normalized relative quantities scaled to control. Error bars indicated the standard deviation (SD) of 3 independent replicates (n = 3). Two-tailed Student t-test was used to compare HaSSP30 *versus* Control in each day and *p-values* < 0.05 are indicated above the bars.

The expression levels of several genes related to the activation of the plant immune system in *N. benthamiana* were assessed by qPCR. The expression of endochitinase B and PI1 (proteinase inhibitor 1) genes showed a significant induction at 2 dpi and 3 dpi, respectively (Fig. 4). The two transcription factors ERF1 (ethylene response factor 1) and WRKY12 were also strongly induced at 1 dpi and 2 dpi, respectively (Fig. 4). However, for both transcription regulators, the gene expression decreased at 3 dpi to a level comparable to the control (Fig. 4). Among the selected pathogenicity related (PR) proteins, only PR3 and PR4a were induced at 3 dpi compared to the control (Fig. 4). PR1a, PR2, and PR5 did not show any significant transcriptional change when compared to the control during the 3-day time course of the experiment (Fig. 4).

## Discussion

*H. annosum* is considered one of the most destructive necrotrophic pathogens of conifers in the northern hemisphere^1^. To date, there is very little information available about the pathogenicity factors that enable this fungus to actively attack and colonize the host. Moreover, there are no data currently available regarding small secreted protein (HaSSP)-encoding genes, which may be important for the fungal virulence. Our approach was aimed to identify candidate HaSSPs that function specifically during the necrotrophic fungus growth stage. Most of the studies about necrotrophic small secreted protein encoding genes are related to crop pathogens, in particular, *S. nodorum* and *P. tritici-repentis* ^22–24^. Additionally, there is basically no information available for necrotrophic forest fungal pathogens, with the exception of the hemibiotroph pine pathogen *Dothistroma septosporum* ^19, 25^. By applying a bioinformatics pipeline approach, we selected 58 putative small secreted protein encoding genes from the *H. annosum* TC 32-1 genome (Table 1). All the selected proteins were presumably capable of being secreted and did not contain any predicted protein domains. The selected HaSSPs are relatively small (Fig. 1), contain disulfide bridges which stabilize the protein structure, and are species-specific with no orthologues in other species^8^. The GEO database contains gene expression data for only 52 HaSSPs out of the 58 selected in this study (Fig. 2). The microarray oligonucleotide probes were selected using the first version of the *H. annosum* TC 32-1 genome annotation (v1.0), while in this study, the HaSSP-encoding genes were selected from the latest genome annotation (v2.0). The missing transcriptomic data for 6 HaSSP-encoding genes in the microarrays can be explained by the differences between the two versions of the genome annotation. However, most of the HaSSP-encoding genes show a high transcript level, indicating that they represent genuine genes that are actively transcribed in *H. annosum*. The 2 conditions related to the necrotrophic growth of *H. annosum* in the pine phloem and xylem form a distinct cluster within the samples. This probably indicates that most of the HaSSP-encoding genes are differentially regulated during the necrotrophic fungal infection compared to the other conditions, which mainly correspond to a saprotrophic lifestyle and growth in liquid media (Fig. 2).

The 58 genes were then synthesized to expedite the screening process and to optimize the codon usage for the Golden Gate cloning approach^26^. Due to the lack of a suitable transformation system in *H. annosum* and to the technical limitations of using mature pine trees for functional gene studies, we selected the well-established *N. benthamiana*/Agrobacterium system to assess the effect of the 58 *H. annosum* HaSSP-encoding genes. *N. benthamiana* has been extensively studied as a model system for elucidating the function and localization of fungal effectors by agroinfiltration^27, 28^. In our study, we found that 8 of the 58 selected HaSSPs from the *H. annosum* genome were able to induce chlorotic lesions and cell death when transiently expressed in *N. benthamiana* (Fig. 3). In a recent study of the rust poplar pathogen *Melampsora larici-populina*, while showing clear subcellular localization of fungal effectors, the authors did not report the induction of plant cell death^27^. However, recent studies investigating the function of selected effectors of the wheat pathogen *Zymoseptoria tritici* and the rice smut pathogen *Ustilaginoidea virens* reported the triggering of plant cell death when these effectors were transiently expressed in the non-host *N. benthamiana* ^29, 30^. Although *H. annosum* has not been previously shown to infect and colonize *N. benthamiana*, we identified 8 HaSSPs that could reproducibly trigger plant cell death. A similar result, both in the number of positive candidates and in the experimental methods, has been achieved for the wheat pathogen *Z. tritici*, for which 14 out of 63 candidate effectors tested were able to induce cell death in the non-host *N. benthamiana* ^29^. While most of the positive candidates could induce a certain level of chlorosis within 3 to 6 dpi, 2 HaSSPs (HaSSP30 and HaSSP47) could strongly induce plant cell death at 2 dpi (Fig. 3). A more detailed sequence comparison between the HaSSP30 and HaSSP47 protein sequences revealed that they are indeed two different isoforms of the same protein (HaSSP47 includes an additional 45 amino acids in its C-terminal domain, Supplementary Fig. S3). This further serves as an internal control that supports the hypothesis that HaSSP30 is a prime candidate for future functional studies.

We investigated the ability of HaSSP30 to induce the activation of the *N. benthamiana* immune system by assessing the gene expression of selected pathogenesis-related genes (PR) and markers for the hypersensitive response (HR). HaSSP30 could induce several Nicotiana chitinase genes (PR3, PR4a, and endochitinase B) at 2 to 3 dpi compared to the control. In contrast, other pathogenesis-related genes (PR1a, PR2, and PR5) did not show any variation in their transcript level compared to the control. The induction of genes belonging to the PR1 and PR2 class was shown to require the plant hormone salicylic acid, which controls the response to biotrophic pathogens^31^. On the other hand, the induction of genes of the PR3 and PR4 class is stimulated by jasmonic acid, which triggers the response to necrotrophic pathogens^31, 32^. Since *H. annosum* is a necrotrophic pathogen, our data suggested that the HaSSP30 protein may act as a necrotrophic effector that triggers a necrotrophic-specific response in plant cells. In an earlier study, we revealed that the defence mechanism against *H. annosum s.l*. in Norway spruce may involve jasmonate-dependent, salicylate-independent signalling^33^. The robust induction of the transcription regulator ERF1 provides further support for the role of HaSSp30 as a potential necrotrophic effector of Nicotiana (Fig. 4). ERF1 acts as a key player in the crosstalk between the plant hormone ethylene and jasmonate signalling by regulating the expression of defence-related genes^34^. In the plant model system *Arabidopsis thaliana*, the transcription of ERF1 was shown to be synergistically activated by both ethylene and jasmonate signalling pathways, which are required for the plant immune response against necrotrophic pathogens^34, 35^. Two other markers for plant cell death, the protease inhibitor PI1 and transcription regulator WRKY12, were also strongly induced in the Nicotiana leaves transiently expressing HaSSP30. The strong induction of PI1 in our study confirmed that the observed symptoms are indeed related to plant cell death, since plant protein inhibitors have been shown to be implicated in defence against pathogens and programmed cell death^36^. On the other hand, WRKY12 is a transcription regulator that has been implicated in the defence against soft rot disease caused by the necrotroph *Pectobacterium carotovorum* in both Arabidopsis and Chinese cabbage^37^. WRKY12 overexpression reduced the disease symptoms and increased the expression of defence genes in *Brassica rapa* L. ssp*. pekinensis*, including PR4, which was also induced in our study^37^. Several WRKY transcriptional regulators have been shown to be important for resistance against necrotrophic pathogens. For example, the Arabidopsis WRKY33 mutation induced increased susceptibility to the necrotrophs *Botrytis cinerea* and *Alternaria brassicicola* ^38^. In conclusion, the transient expression in *N. benthamiana* of HaSSP30 alone can trigger a transcriptional response, which is consistent with previous reports of necrotrophic pathogens.

In this study, we demonstrated the feasibility of the use of the *N. benthamiana* agroinfiltration system to identify putative effector-like proteins from the conifer necrotrophic pathogen *H. annosum*. Although *Heterobasidion* has not been previously shown to infect Nicotiana plants, our results revealed that transient expression of certain predicted HaSSPs in this system could induce plant cell death. The mechanism of action is still unclear, especially with regard to the hypothetical protein HaSSP30. However, we hypothesize that similar activation pathways for the host immune system may be conserved between Nicotiana and conifers. In this case, HaSSP30 could potentially interact with its Nicotiana orthologues and, consequently, trigger cell death. The identification of the specific plant receptors that interact with the cell death-inducing HaSSP30 will be the next research priority. The elucidation of the mechanism of action of HaSSP30 in Nicotiana will shed light on the *H. annosum* infection mechanisms in natural conifer hosts.

## Materials and Methods

### Bacterial, fungal strains and growth conditions

*Escherichia coli* TOP10F (Thermo Fisher Scientific, Finland) was used for gene cloning and plasmid propagation. Expression plasmids were introduced in *Agrobacterium tumefaciens* GV3101 competent cells by cold shock protocol. *Nicotiana benthamiana* was used for the transient gene expression and quantitative PCR (qPCR) experiments. The plants were grown at 12 h/12 h, night/day photoperiods at 20–24 °C with 60% relative humidity.

### Selection of the candidate small secreted protein (HaSSPs) encoding genes

The sequenced genome of *H. annosum* TC32-1, available in Mycocosm^39^ at the JGI portal (http://genome.jgi.doe.gov/Hetan2/Hetan2.home.html, v2.0 June 2010), was used to identify the putative small secreted protein (HaSSP) encoding genes investigated in this study^21^. The pipeline used is a modified version of the one previously described^40^. Briefly, the PexFinder program^41, 42^ was used to identify all the proteins with signal peptides from the list of all predicted proteins in the *H. annosum* TC 32-1 genome. The list was then condensed by removing transmembrane, mitochondrial, and nuclear proteins using TMHMM (http://www.cbs.dtu.dk/services/TMHMM/), TargetP^43^, and PredictNLS^44^. Additionally, proteins with a predicted InterPro domain and wood-degrading enzymes were removed in order to focus only on novel and uncharacterized HaSSPs. Finally, we predicted disulfide bridges, which are thought to stabilize the protein structure, using DiANNA^45^.

### Analysis of the expression levels of the candidate *H. annosum* small secreted protein (HaSSP) encoding genes from microarray data

Transcriptomic data related to several fungal growth conditions were used to verify the expression levels of the candidate HaSSPs. The *H. annosum* transcriptomic data were retrieved from the public GEO database^46^. The dataset contains the transcriptional response of *H. annosum* grown under different conditions: necrotrophic growth in pine, saprotrophic growth on wood material and soil, and growth in various liquid media. The details of the experimental conditions, RNA extraction, and microarray hybridization were as previously described^21, 47^. Data GEO accession numbers, description of the samples, and reference to the publications are summarized in Supplementary Table S1.

The microarray raw data were imported in R^48^, and the *oligo* package^49^ was used to normalize and extract the microarray expression values for the selected HaSSPs. For each gene, the mean value for the all biological replicates was calculated to have one single transcriptional profile for each condition. The cluster analysis was performed with the *gplots* package using the Euclidean distance to calculate the distance matrix and the average method for the agglomeration clustering^50^. Finally, the statistic *p-values* for the sample and gene clusters were calculated in R using the *pvclust* package to assess the statistical significance of the cluster analysis^51^.

### Cloning of the candidate *H. annosum* small secreted protein (HaSSP) encoding genes

The genes encoding the selected candidate *H. annosum* small secreted protein (HaSSP) were custom synthesized (GeneWiz, UK). Briefly, the restriction sites for the type II enzymes *Bpi*I and *Bsa*I were removed from the gene coding sequence by codon optimization (GeneWiz, UK). The nucleotide sequences were designed to include *Bpi*I restriction sites at both termini (5′-CACCGAAGACACAATG/TAAGCTTCCGTCTTCGTAG-3′) and cloned into pUC57 plasmid. The HaSSP coding sequences were then cloned by the Golden Gate approach into the Level0 pICH41308 plasmid (Addgene, UK)^26, 52^ using *Bpi*I (NEB, Finland). From Level0 plasmids, the HaSSP coding sequences were cloned into the expression vector, Level2 pICH86988, creating the final vector pICH86988-HaSSP (35 S promoter and octopine synthase terminator, Addgene, UK) using the *Bsa*I restriction enzyme (NEB, Finland). All cloning procedures were performed using the Golden Gate approach (for details about Golden Gate cloning visit http://synbio.tsl.ac.uk/golden-gate/) and *E. coli* TOP10F for selection of positive transformants.

### Transient expression of the candidate *H. annosum* small secreted protein (HaSSPs) encoding genes in *Nicotiana benthamiana*

The pICH86988-HaSSP vector was transferred to *A. tumefaciens* GV3101 by the cold-shock transformation method. The bacteria were selected on LB solid media containing gentamicin (100 µg/ml), rifampicin (10 µg/ml), tetracycline (3 µg/ml), and kanamycin (50 µg/ml). For agroinfiltration in *N. benthamiana*, the *A. tumefaciens* carrying the selected construct to be expressed was grown in 3 ml liquid LB media supplemented with gentamicin (100 µg/ml), rifampicin (10 µg/ml), tetracycline (1.5 µg/ml), and kanamycin (20 µg/ml) overnight at 28 °C under shaking. The day after, 10–20 µl of the LB culture were inoculated into 10 ml YEB medium (5 g/l beef extract, 1 g/l yeast extract, 5 g/l bacteriological peptone, 5 g/l sucrose, and 2 ml of 1 M MgSO_4_) containing gentamicin (100 µg/ml), rifampicin (10 µg/ml), tetracycline (1.5 µg/ml), kanamycin (20 µg/m), 2 μM acetosyringone, and 10 mM MES. The cultures were grown overnight at 28 °C and 200 rpm to an OD_600_ = 1. The bacteria were harvested by centrifugation (1500 *g* for 5 min) and resuspended in 5 ml MMA infiltration medium (5 g/l MS salts, 1.95 g/l MES (2-[N-Morpholino] ethane sulfonic acid), 20 g/l sucrose, pH = 5.6) supplemented with 200 μM acetosyringone. The bacterial liquid culture was finally adjusted to OD_600_ = 0.3 with MMA infiltration medium and incubated in the dark at room temperature for 2 h. *N. benthamiana* leaves were agroinfiltrated with the *A. tumefaciens* carrying the selected pICH86988-HaSSP and incubated until symptoms developed in 2 to 6 days.

### Assessment of the expression levels of plant immune response-related genes in *Nicotiana benthamiana* infiltrated with the HaSSP30 gene

*N. benthamiana* leaves were infiltrated with *A. tumefaciens* carrying either pICH86988-empty (Control) or pICH86988-HaSSP30 (HaSSP30) with 3 biological replicates each. Total RNA was extracted after 1, 2, and 3 days post infiltration (dpi) using TRI Reagent (Sigma-Aldrich, Finland) following the manufacturer’s instructions. Total RNA was reverse transcribed as follows: 1 µg RNA was treated with DNase I (Thermo-Fisher Scientific, Finland) at 37 °C for 30 min followed by enzyme inactivation by adding 1 µl EDTA 50 mM at 65 °C for 10 min in 11 µl total volume. One microliter of random hexamer primers (100 µM) and 0.5 µl nuclease free water were added to the DNase treated RNA; the reaction was incubated at 65 °C for 5 min and then cooled on ice. The following components were then added to the reaction mixture: 2 µl dNTPs (10 mM), 0.5 µl RiboLock RNase Inhibitor, 4 µl 5X RevertAid Reverse Transcriptase buffer, and 1 µl RevertAid Reverse Transcriptase (200 U/µl) (Thermo-Fisher Scientific, Finland). The RNA was reverse transcribed by incubating the reaction mixture for 10 min at 25 °C and then for 60 min at 42 °C. The reaction was finally incubated at 70 °C for 5 min. The final volume was 20 µl.

Nine markers for the plant immune system (Endochitinase B, ERF1a, PI1, PR1a, PR2, PR3, PR4a, PR5, and WRKY12) and 2 reference genes (EF1a and Actin) were assessed by qPCR. The qPCR reaction mixture was assembled with 5.5 µl cDNA template (1:15 dilution), 1 µl forward primer, 1 µl reverse primer, and 7.5 µl 2X LightCycler 480 SYBR Green I Master (Roche, Finland). The 384-well plates were analysed via a LightCycler 480 II (Roche, Finland) with the following program: preincubation at 95 °C for 5 min, followed by 45 amplification cycles (95 °C for 10 sec, 60 °C for 10 sec, and 72 °C for 10 sec). Following cycle completion, samples were subjected to melting curve analysis to assess the primer specificity. The qPCR data were analysed in R^48^ using the EasyqpcR package^53^ and normalized using 2 reference genes (EF1a and Actin). Primer information is summarized in Supplementary Table S2.

## Electronic supplementary material

Supplementary Figures
