# Supplementary material for: Small secreted proteins from the necrotrophic conifer pathogen Heterobasidion annosum s.l. (HaSSPs) induce cell death in Nicotiana benthamiana
Source: Sci Rep. 2017 Aug 11;7:8000. doi: 10.1038/s41598-017-08010-0 (PMC5554239; doi:10.1038/s41598-017-08010-0)
Supplement: Supplementary file 1 — Supplementary Figures [file 41598_2017_8010_MOESM1_ESM.pdf]

Small secreted proteins from the necrotrophic conifer pathogen *Heterobasidion annosum s.l.* (HaSSPs) induce cell death in *Nicotiana benthamiana*

Tommaso Raffaello & Fred O. Asiegbu

Supplementary Figure 1: Statistical analysis of the microarray sample data clustering by multiscale bootstrap resampling. Microarray data were retrieved from the Gene Expression Omnibus (GEO) database (https://www.ncbi.nlm.nih.gov/geo/). Raw data were normalized with the statistical program R[48] using the *oligo* package[49]. The statistic *p-values*, "au" (approximately unbiased) and "bp" (bootstrap probability), for the sample clusters were calculated in R using the *pvclust* package in order to assess the statistical significance of the cluster analysis by multiscale bootstrap resampling[51].

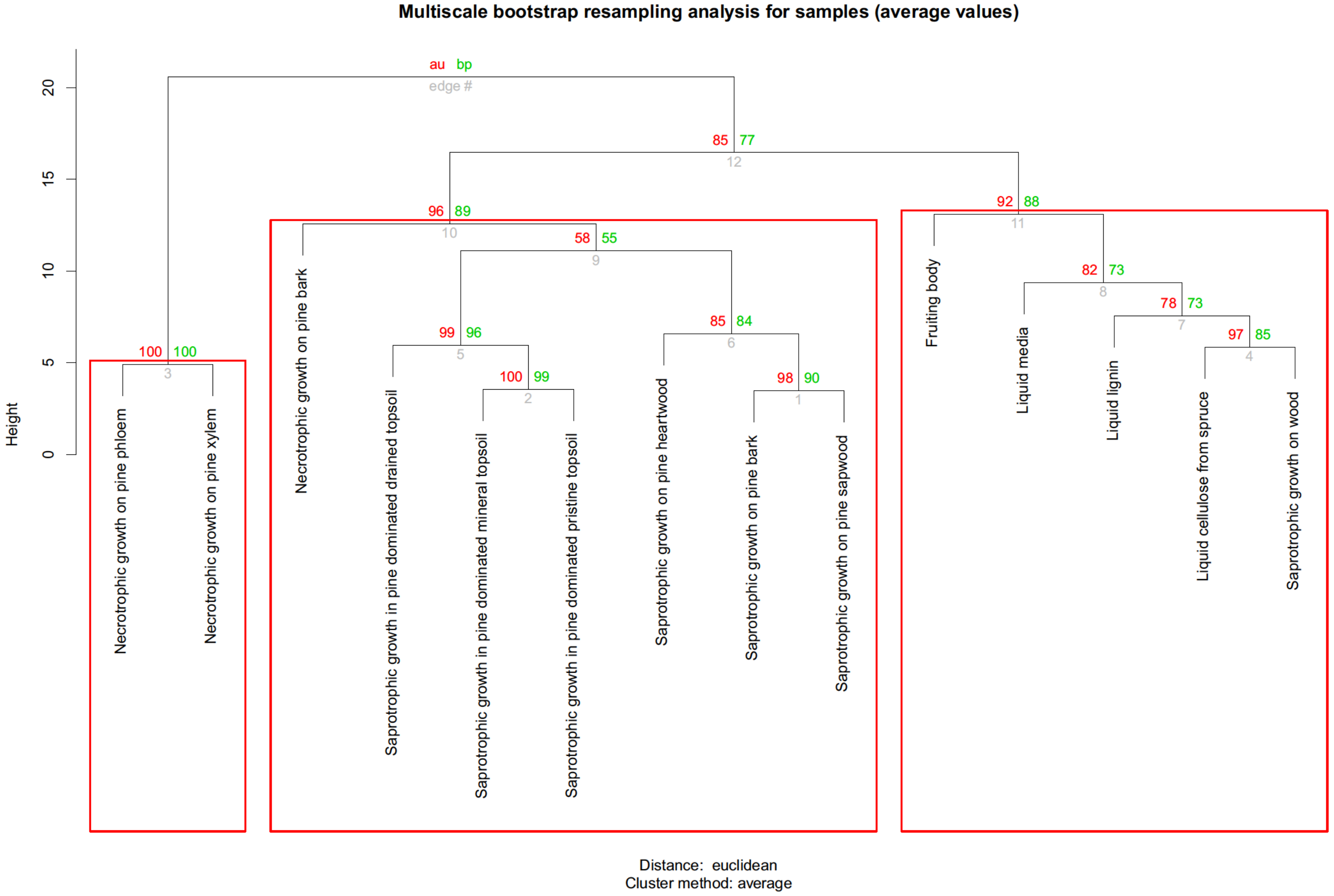

Supplementary Figure 2: Statistical analysis of the microarray gene data clustering by multiscale bootstrap resampling. Microarray data was retrieved from the Gene Expression Omnibus database (GEO) (https://www.ncbi.nlm.nih.gov/geo/). Raw data was normalized with the statistical program R[48] using the *oligo* package[49]. The statistic *p-values*, “au” (approximately unbiased) and “bp” (bootstrap probability), for the gene clusters were calculated in R using the *pvclust* package in order to assess the statistical significance of the cluster analysis by multiscale bootstrap resampling[51].

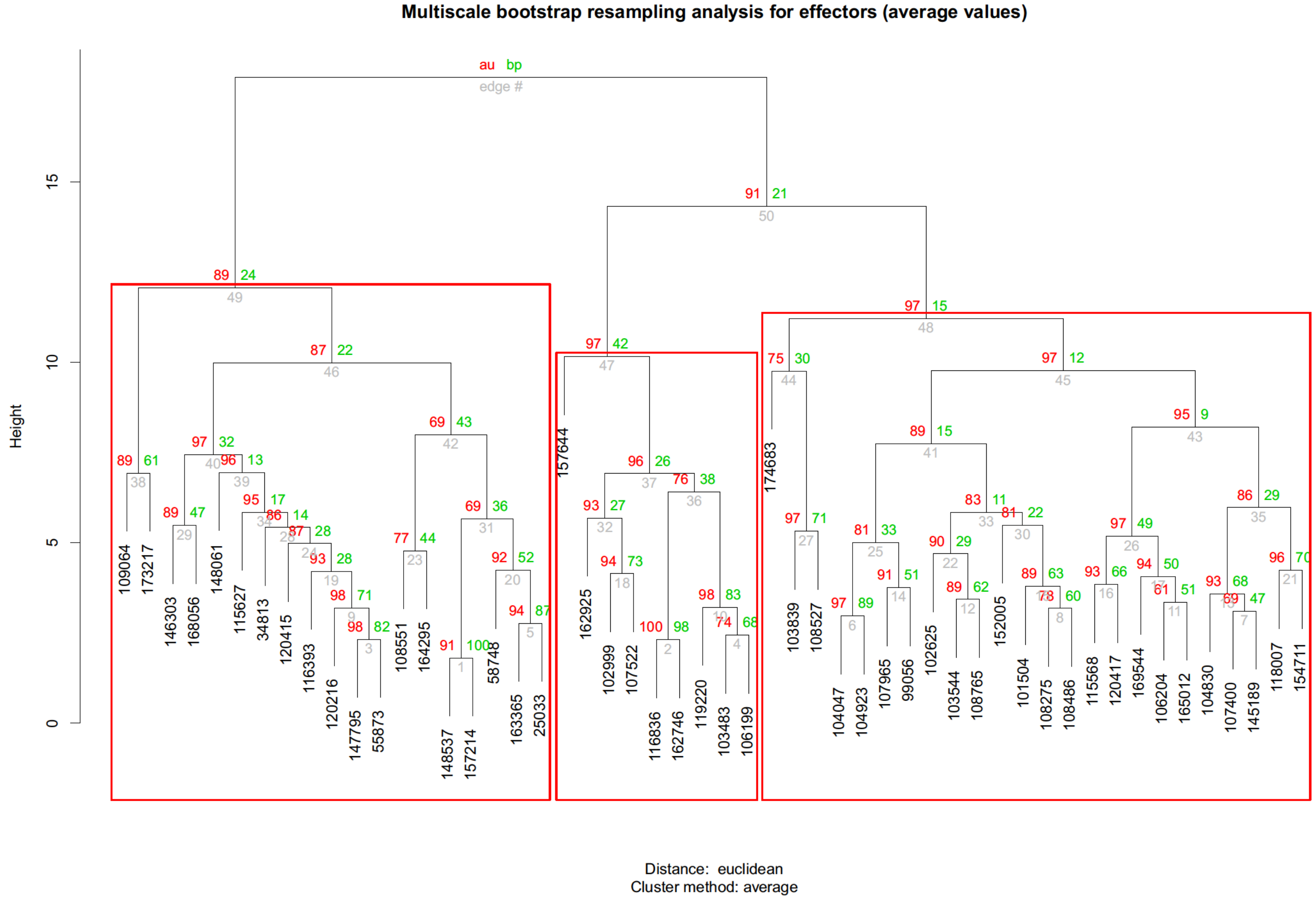

Supplementary Figure 3: Comparison of HaSSP30 and HaSSP47 protein length.

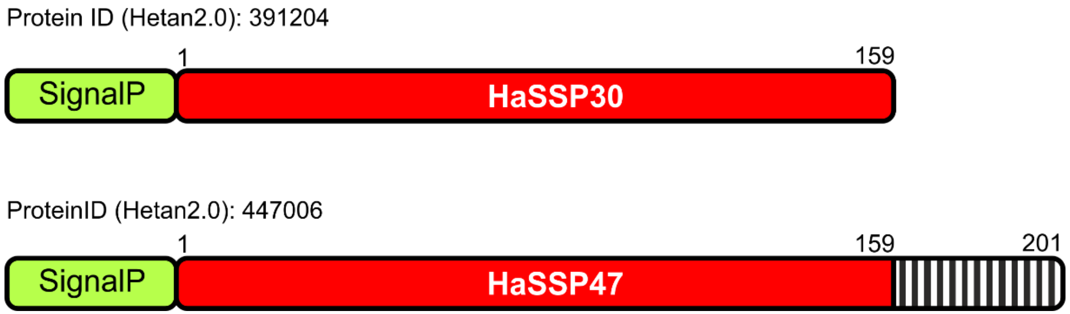

Supplementary Table S1: List of the microarray samples analysed in this study from the Gene Expression Omnibus database (GEO).

Accession number in Gene Expression Omnibus (GEO) https://www.ncbi.nlm.nih.gov/geo/	Sample description[1]	Reference
**GSM748496**	necrotic bark of pines inoculated with Heterobasidion irregulare 1	(Olson, Aerts et al. 2012)
**GSM748497**	necrotic bark of pines inoculated with Heterobasidion irregulare 2	(Olson, Aerts et al. 2012)
**GSM748498**	necrotic bark of pines inoculated with Heterobasidion irregulare 3	(Olson, Aerts et al. 2012)
**GSM748499**	Heterobasidion irregulare grown in liquid medium amended with cellulose from Spruce 1	(Olson, Aerts et al. 2012)
**GSM748500**	Heterobasidion irregulare grown in liquid medium amended with cellulose from Spruce 2	(Olson, Aerts et al. 2012)
**GSM748501**	Heterobasidion irregulare fruiting bodies collected in California 1	(Olson, Aerts et al. 2012)
**GSM748502**	Heterobasidion irregulare fruiting bodies collected in California 2	(Olson, Aerts et al. 2012)
**GSM748503**	Heterobasidion irregulare fruiting bodies collected in California 3	(Olson, Aerts et al. 2012)
**GSM748504**	Heterobasidion irregulare fruiting bodies collected in California 4	(Olson, Aerts et al. 2012)
**GSM748505**	H. irregulare grown on wood shavings from pine 1	(Olson, Aerts et al. 2012)
**GSM748506**	H. irregulare grown on wood shavings from pine 2	(Olson, Aerts et al. 2012)
**GSM748559**	H. irregulare grown on wood shavings from pine 3	(Olson, Aerts et al. 2012)
**GSM748560**	H. irregulare grown on wood shavings from pine 4	(Olson, Aerts et al. 2012)
**GSM748572**	Heterobasidion irregulare grown in liquid medium amended with lignin 1	(Olson, Aerts et al. 2012)
**GSM748573**	Heterobasidion irregulare grown in liquid medium amended with lignin 2	(Olson, Aerts et al. 2012)
**GSM979475**	H. annosum grown on pulverized Scots pine bark shavings, rep1	(Raffaello, Chen et al. 2014)
**GSM979476**	H. annosum grown on pulverized Scots pine bark shavings, rep2	(Raffaello, Chen et al. 2014)
**GSM979477**	H. annosum grown on pulverized Scots pine bark shavings, rep3	(Raffaello, Chen et al. 2014)
**GSM979481**	H. annosum grown on liquid malt extract medium, rep1	(Raffaello, Chen et al. 2014)
**GSM979482**	H. annosum grown on liquid malt extract medium, rep2	(Raffaello, Chen et al. 2014)
**GSM979483**	H. annosum grown on liquid malt extract medium, rep3	(Raffaello, Chen et al. 2014)
**GSM979490**	H. annosum grown on pulverized Scots pine heartwood shavings, rep1	(Raffaello, Chen et al. 2014)
**GSM979492**	H. annosum grown on pulverized Scots pine heartwood shavings, rep2	(Raffaello, Chen et al. 2014)
**GSM979494**	H. annosum grown on pulverized Scots pine heartwood shavings, rep3	(Raffaello, Chen et al. 2014)
**GSM979515**	Necrotic phloem of Scots pine infected with H. annosum, rep1	(Raffaello, Chen et al. 2014)
**GSM979516**	Necrotic phloem of Scots pine infected with H. annosum, rep2	(Raffaello, Chen et al. 2014)
**GSM979517**	Necrotic phloem of Scots pine infected with H. annosum, rep3	(Raffaello, Chen et al. 2014)
**GSM979530**	H. annosum grown on pulverized Scots pine sapwood shavings, rep1	(Raffaello, Chen et al. 2014)

Supplementary Table S1 (*Continued*)

**GSM979531**	H. annosum grown on pulverized Scots pine sapwood shavings, rep2	(Raffaello, Chen et al. 2014)
**GSM979532**	H. annosum grown on pulverized Scots pine sapwood shavings, rep3	(Raffaello, Chen et al. 2014)
**GSM979536**	Necrotic xylem of Scots pine infected with H. annosum, rep1	(Raffaello, Chen et al. 2014)
**GSM979537**	Necrotic xylem of Scots pine infected with H. annosum, rep2	(Raffaello, Chen et al. 2014)
**GSM979538**	Necrotic xylem of Scots pine infected with H. annosum, rep3	(Raffaello, Chen et al. 2014)
**GSM1333665**	H. annosum grown on Pine-dominant mineral forest topsoil (organic layer) 1	Unpublished
**GSM1333666**	H. annosum grown on Pine-dominant mineral forest topsoil (organic layer) 2	Unpublished
**GSM1333667**	H. annosum grown on Pine-dominant mineral forest topsoil (organic layer) 3	Unpublished
**GSM1333668**	H. annosum grown on Pine-dominant pristine peatland forest topsoil (organic layer) 1	Unpublished
**GSM1333669**	H. annosum grown on Pine-dominant pristine peatland forest topsoil (organic layer) 2	Unpublished
**GSM1333670**	H. annosum grown on Pine-dominant pristine peatland forest topsoil (organic layer) 3	Unpublished
**GSM1333671**	H. annosum grown on Pine-dominant drained peatland forest topsoil (organic layer) 1	Unpublished
**GSM1333672**	H. annosum grown on Pine-dominant drained peatland forest topsoil (organic layer) 2	Unpublished
**GSM1333673**	H. annosum grown on Pine-dominant drained peatland forest topsoil (organic layer) 3	Unpublished

[1]Biological replicates are indicated by the Arabic number at the end of the description.

Supplementary Table S2: List of primers used in this study.

Gene	Sequence used to design PCR and qPCR primers	Forward primer	Reverse primer	Amplicon length	E	Reference
PR1a	NA	CGACCAGGTAGCAGCCTATG	TCTCAACAGCCTTAGCAGCC	NA	2	Qi M, et al., 2016
PR2	NA	GGGCTGTTAATTTGCAGTATCC	GGTTTATAACATCTTGGTCTGATGG	NA	2	Qi M, et al., 2016
WRKY12	NA	CTCATCAGCTAGTTCATTTGATGC	AGCTCGGTCTTTGTTCTAAAAGC	NA	2	Qi M, et al., 2016
PI1	NA	CTTCAAAGACTATGGTGAAGTTTGC	CAGACTGAGACACATCAAGTTGC	NA	2	Qi M, et al., 2016
PR3	>PR-3<br>ATGGAGTTTTCTGGATCACCACTGGTATTGTTTTGTTGTGTGTTTTTCCTGTTCT<br>TAACAGGGAGCTTGGCACAAGGCATTGGTTCTATTGTGACGAGTGACTTGTTC<br>AACGAGATGCTGAAGAATAGGAATGACGTTAGATGTCCTGCCAATGGCTTCTA<br>CACTTATGATGCATTCATAGCTGCTGCCAATTCCTTTCCTGGTTTTGGAACTACT<br>GGTGATGATACTGCCCGTAGGAAAGAAATTGCTGCCTTTTTCGGTCAAACTTCT<br>CATGAAACTACTGGTGGTTCCCTGAGTGCAGAACCATTTACAGGAGGATATTG<br>CTTTGTTCGGCAAAATGACCAGAGTGAAAGATATTATGGTAGAGGACCCATCC<br>AATTGACAAACCGAAATAATTATGAGAAAGCTGGAACTGCAATTCAACAAGAC<br>CTAGTTAACAACCCTGATTTAGTAGCCACAGATGCTACTATATCATTCAAAACA<br>GCTATATGGTTTTGGATGACAGCACAGGACAACAAGCCATCTTCCCACGACGT<br>TATCATCGGTAGTTGGACTCCGTCTGCCGCGGATCAGGCGGCGAATCGAGTAC<br>CAGGTTACGGTGTAATTACCAACATCATTAACGGTGGAATTGAATGTGGCATG<br>GGTCGAAATGACGCAGTGGAAGATCGAATTGGATACTACAGGAGGTATTGTG<br>GAATGTTAAATGTTGCTCCTGGGGAAAACTTGGACTGTTACAACCAAAGGAAC<br>TTCGCCCAGGGCTAG	TGCCTTTTTCGGTCAAACTT	TGTAAATGGTTCTGCACTCAGG	64	1.87	This study
PR4a	>PR-4a<br>ATGGAGAGAGTAAATAATTACTATAAGTTGTGTATGGAATTGTTTATCATGAG<br>CATGATGGTGGCAATGGCGGCGGCACAGAGCGCTACAAACGTGAGATCAACG<br>TATCATTTATACAACCCACAGAACATTAACTGGGATTTGAGAGCAGCAAGTGC<br>TTTCTGCACTACTTGGGATGCCGACAAGCCTCTCACGTGGCGTCAGAAATATG<br>GCTGGACTGCTTTCTGTGATGCTGCTGGACCTCAAGGCCAAGATTCCTGTGGT<br>AGATGCTTGAGGGTGACGAACACAGGAACAGGAACTCAAACAACAGTGAGAA<br>TAGTAGATCAATGCAGAAATGGAGGGCTTGATTTGGATGTAAACGTCTTTAAC<br>CAATTGGACACAAATGGAGTGGGCTATCAGCAAGGCCACCTTATTGTCAACTA<br>TGAATTTATCAACTGCGATGACTAA	CAACCCACAGAACATTAACTGG	TTGTCGGCATCCCAAGTAGT	69	1.9	This study
PR5	>PR-5<br>ATGAACTTCCTCAAAAGCTTCCCCTTTTTTGCCTTCCTTTGTTTTGGCCAATACTT<br>TGTAGCTGTTACTCATGCTGCAACTTTTGACATTATCAACCAATGCACCTACAC<br>AGTCTGGGCCGCGGCCTCTCCAGGTGGAGGCAGGCAGCTCAACTCGGGCCAA<br>TCTTGGAGCATTAACGTGAACCCAGGAACAGTCCAGGCTCGCATATGGGGCC<br>GAACCAACTGCAACTTCGATGGCAGTGGCCGAGGTAATTGTGAGACTGGAGA<br>CTGTAACGGGATGCTAGAGTGTCAAGGCTATGGTAAACCACCCAACACTTTAG<br>CTGAATTTGCACTTAATCAGCCTAATCAGGACTTCGTCGACATCTCTCTTGTTGA<br>TGGATTTAACATCCCCATGGAATTCAGCCCAACTAATGGCGGGTGCCGTAACC<br>TTAGATGCGCAGCCCCTATTAACGAGCAATGCCCAGCACAGTTGAAAACACAA<br>GGTGGATGTAACAACCCATGTACTGTGATAAAAACCAATGAATATTGTTGTAC<br>AAATGGGCCTGGATCATGTGGGCCTACTGATTTGTCGAGATTTTTTAAGGAAA<br>GATGCCCAGATGCTTATAGCTATCCACAGGATGATCCAACTAGTTTGTTTACTT<br>GTCCTTCTGGTACCAATTACAGGGTTGTCTTCTGCCCTTGA	ATGCGCAGCCCCTATTAAC	TGGGTTGTTACATCCACCTTG	67	1.88	This study

Supplementary Table S2 (*Continued)*

Gene	Sequence used to design PCR and qPCR primers	Forward primer	Reverse primer	Amplicon length	E	Reference
ERF1	>ERF1<br>ATGGGCTCACCACAAGAGAATTGCACTACACTTGATTTAATTAGGCAACATCTTCTTGATGATAATGTTTTCATGG<br>AACATTATTGTCCCCAACCAATTCTTTATTCTCAAAGCTCCTCCTCTTCTGAATCTTTAAACTCCATTGCTTCTGAGC<br>TCAATAACGATAGTTTCTCCTTTGAACCAACTCTCAATTATGCCGACACAGCCCAAAGTTCCAATCTTGATATCTCA<br>ACCTTCTTTAACAATTCAAAAACAGAGTTCGACTGCTTTGAGTTTGAGACAAAACCAAACGTGTTAGCTGCTCGTA<br>TTAGTTCAAATTCTCCGAAGCAAACAAGCTTCAACGAACGCAAGCCTTCTCTGAATATTGCTATACCCCTGAAGCA<br>GCAAGAGGTTGTTCAGAAAGTGGGAATTTCCAACGAGAGCAAGAAGAAGCATTACCGAGGAGTTAGGCAACGG<br>CCGTGGGGGAAGTTCGCCGCAGAGATTCGTGACCCGAACCGAAAGGGGACTCGGGTTTGGTTAGGAACCTTTG<br>ACACTGCCTTAGAGGCGGCTAAGGCATATGATAGGGCGGCGTTTAAGCTTAGAGGTAGCAAAGCAATAGTTAAT<br>TTCCCTCTCGAAGTTTCAAACTTTAAGCAACAAGATAATGAGATTCAGCCTTTGATGAACTCAAACAGGAAAAGG<br>GTGAGAGAAACAGAGAGTGAGGAGCAACTAGTTGTCGACAAGGAAATGAAAATTGAACAAGAAAGAGTCCCAA<br>CAGCTCCGTTAACGCCGTCAAGTTGGTCGGCGATTTGGGACAGTGGAAATGGGAAGGGTATATTTGAGGTGCCG<br>CCTCTATCACCATTATCTCCACATATGGCTTATTCTCAGCTTGTCATGATATAA	GTTAACGCCGTCAAGTTGGT	AGAGGCGGCACCTCAAATA	72	1.93	This study
Endochitinase B	> Endochitinase B<br>ATGAGGCTTTGTAAATTCACAGCTCTTTCTTCTCTACTATTTTCTCTCCTATTGCTTTCTGCCTCGGCAGAACAATGT<br>GGTTCGCAGGCCGGAGGTGCGCGTTGTCCCTCGGGTCTCTGCTGCAGCAAATTTGGTTGGTGTGGTAACACCAA<br>TGACTATTGTGGCCCTGGCAATTGCCAGAGCCAGTGCCCTGGTGGTCCCACACCTACACCGCCCACCCCACCCGG<br>TGGTGGGGACCTCGGCAGTATCATTTCAAATTCCATGTTTGATCAGATGCTTAAGCATCGCAACGATAATGCCTG<br>CGAAGGACATGGATTCTACAGTTACAATGCCTTTATCAATGCTGCTAGGTCTTTTCCTGGCTTTGGTACCAGTGGC<br>GATACTACTGCCCGAGGATGGGCAACAGCACCAGATGGTCCATATGCATGGGGTTATTGCTGGCTTAGAGAACG<br>AGGTAGCCCCGGCGACTACTGTACACCAAGTGGTCAGTGGCCTTGTGCTCCTGGTCGCAAATATTTCGGACGAG<br>GCCCCATCCAAATTTCACACAACTATAACTACGGACCTTGTGGAAGAGCCATAGGAGTGGACCTCCTAAACAATC<br>CTGATTTAGTGGCCACAGATCCAGTTATCTCATTTAAGTCAGCTCTCTGGTTTTGGATGACCCCTCAATCACCAAA<br>ACCTTCTTGCCACGATGTCATCATCGGAAGATGGCAACCATCGTCTGCTGACCGCGCAGCCAATCGTCTCCCTGG<br>ATTTGGTGTCATCACGAACATCATCAACGGTGGCCTGGAATGTGGTCATGGCACTGACTCAAGGGTCCAGGATC<br>GCGTTGGGTTTTACAGAAGGTATTGCAGTATTCTTGGAGTTAGTCCTGGTGACAATCTTGATTGCGGCAACCAGA<br>GGTCTTTTGGAAACGGACTTTTAGTCGATACTATGTAA	GCCTTTATCAATGCTGCTAGG	ATCCTCGGGCAGTAGTATCG	67	1.88	This study
EF1a	>EF1a<br>ATGGGTAAAGAAAAAGTTCACATCAACATTGTGGTCATTGGCCACGTCGATTCTGGAAAGTCTACCACCACTGGT<br>CACTTGATCTACAAGCTTGGTGGTATTGACAAGCGTGTTATTGAGAGGTTCGAGAAGGAAGCCGCTGAGATGAA<br>CAAGAGGTCATTCAAGTATGCCTGGGTGCTTGACAAGCTTAAGGCTGAACGTGAGCGGGGTATCACCATTGATA<br>TTGCCTTGTGGAAGTTTGAGACCACCAAATACTACTGCACTGTGATTGATGCCCCCGGACACAGGGACTTTATCA<br>AGAACATGATCACTGGTACTTCCCAGGCTGATTGTGCTGTTCTTATTATTGACTCCACCACTGGTGGTTTTGAAGC<br>TGGTATTTCCAAGGATGGTCAGACCCGTGAACACGCATTGCTTGCTTTCACCCTTGGTGTCAAGCAAATGATTTGC<br>TGCTGCAACAAGATGGATGCTACCACCCCCAAGTACTCCAAGGCTAGGTATGATGAAATCGTCAAGGAAGTTTCT<br>TCCTACCTGAAGAAGGTTGGTTACAACCCTGACAAGATTCCCTTCGTCCCCATCTCTGGTTTTGAGGGAGACAATA<br>TGATTGAGAGGTCTACCAACCTTGACTGGTACAAGGGGCCAACCCTCCTTGAGGCTCTTGACCAGATTAATGAGC<br>CCAAGAGGCCCACAGACAAACCCCTACGTCTTCCACTTCAGGACGTTTACAAGATTGGTGGTATTGGTACCGTCC<br>CCGTTGGTCGTGTTGAAACTGGTGTCCTCAAGCCTGGTATGGTTGTGACCTTTGGGCCTACTGGTCTGACAACTG<br>AAGTCAAGTCTGTAGAGATGCACCACGAAGCTCTCCTGGAGGCACTCCCCGGTGACAATGTTGGGTTCAATGTTA<br>AGAATGTTGCTGTTAAGGATCTCAAGCGTGGTTATGTTGCCTCAAATTCCAAGGATGACCCAGCCAAAGGGGCT<br>GCCAGTTTCACCTCCCAGGTCATCATCATGAACCATCCTGGTCAGATTGGAAATGGATATGCACCAGTTCTTGACT<br>GCCACACTTCCCACATTGCTGTCAAGTTCGCTGAGATCTTGACCAAGATTGACAGACGTTCTGGTAAGGAACTTG<br>AGAAGGAGCCTAAGTTCTTGAAGAATGGTGATGCTGGTATGGTTAAGATGATGCCGACCAAGCCTATGGTTGTG<br>GAGACCTTTGCCGAGTACCCACCATTGGGTCGTTTTGCTGTGAGGGACATGCGTCAGACTGTTGCTGTTGGTGTC<br>ATCAAGAGTGTTGACAAGAAGGACCCAACTGGCGCCAAGGTCACCAAAGCTGCTCAGAAGAAGAAGTGA	CTCCTTGAGGCTCTTGACCA	ACGTAGGGGTTTGTCTGTGG	60	1.91	This study

Supplementary Table S2 (*Continued)*

Gene	Sequence used to design PCR and qPCR primers	Forward primer	Reverse primer	Amplicon length	E	Reference
Actin	>Actin<br>ATGGCAGATGGAGAGGATATTCAGCCACTTGTCTGTGACAATGGAACAGGAATGGTCAAGGCTGG<br>GTTTGCTGGAGATGATGCTCCACGAGCTGTATTCCCTAGTATTGTTGGCCGGCCCCGCCATACTGG<br>TGTGATGGTGGGTATGGGTCAGAAAGATGCCTACGTGGGAGATGAAGCTCAATCAAAAAGAGGT<br>ATTTTAACTCTTAAATACCCAATTGAGCATGGAATTGTCAGCAACTGGGATGATATGGAGAAGATC<br>TGGCATCATACTTTCTACAATGAGCTTCGTGTTGCGCCCGAGGAGCATCCAGTCCTCCTAACTGAAG<br>CGCCTCTTAACCCAAAGGCTAATCGTGAAAAGATGACCCAGATTATGTTTGAGACTTTTAATACCCC<br>AGCTATGTATGTTGCTATTCAGGCTGTCCTCTCACTGTATGCCAGTGGTCGTACCACCGGTATTGTG<br>TTGGACTCTGGTGATGGTGTCAGCCACACCGTCCCAATTTATGAGGGGTATGCCCTCCCACATGCC<br>ATTCTCCGTCTTGACTTGGCAGGCCGTGACCTCACTGATAGTTTGATGAAGATCCTTACCGAGCGT<br>GGTTACATGTTCACCACCTCAGCTGAGCAGGAAATTGTCAGGGACGTGAAAGAAAAGCTTGCTTA<br>CATAGCTCTTGACTATGAACAGGAACTCGAGACTGCAAAGACCAGCTCTTCTGTAGAGAAGAACTA<br>TGAGCTCCCAGATGGGCAGGTGATCACCATTGGTGCTGAGCGTTTCCGTTGTCCTGAGGTCCTTTT<br>CCAACCATCAATGATTGGAATGGAAGCTGCAGGAATCCACGAGACTACATACAACTCTATCATGAA<br>GTGTGATGTGGATATTAGAAAGGATCTTTATGGAAACATTGTGCTCAGTGGTGGCTCTACCATGTT<br>CCCGGGTATCGCTGATAGAATGAGCAAAGAAATTACTGCGCTTGCTCCTAGCAGCATGAAGATTAA<br>GGTGGTGGCCCCACCAGAGAGGAAATACAGTGTCTGGATTGGAGGCTCTATCTTGGCTTCCCTCA<br>GCACCTTCCAGCAGATGTGGATTGCTAAGGCAGAGTATGACGAATCTGGTCCGTCTATTGTCCACA<br>GAAAGTGCTTCTGA	TACCACCGGTATTGTGTTGG	TCATAAATTGGGACGGTGTG	60	1.82	This study
